# The Assessment of Functional Status Among COVID-19 Patients at Three Months Using Pulmonary Function Tests

**DOI:** 10.7759/cureus.61221

**Published:** 2024-05-28

**Authors:** Mohini Asija, Shaveta Dahiya, Rohit Parsad, Sanjay Fotedar, Rohit Sharma, Vikas Bhatthi

**Affiliations:** 1 Internal Medicine, Pandit Bhagwat Dayal Sharma Post Graduate Institute of Medical Sciences, Rohtak, IND; 2 General Medicine, Pandit Bhagwat Dayal Sharma Post Graduate Institute of Medical Sciences, Rohtak, IND

**Keywords:** six-minute walk test, spirometry, follow-up, functional limitation, post-covid-19 symptoms

## Abstract

Introduction

The coronavirus disease 2019 (COVID-19) pandemic has impacted the lives of thousands of patients worldwide with many patients having residual symptoms months after the acute infection. The severity of lung involvement ranges from mild asymptomatic to severe acute respiratory distress syndrome (ARDS), which may lead to pulmonary fibrosis. Pulmonary fibrosis increases the long-term morbidity of post-COVID-19 patients in the form of restrictive lung disease. The six-minute walk test (6MWT), Borg scale, and spirometry are simple and low-cost tests used to evaluate a patient's exercise capacity and functional status. This study was conducted to assess the residual symptoms and functional status using spirometry and 6MWT in COVID-19 patients of moderate to severe category after three months of discharge.

Methods

This was an observational, prospective, and cross-sectional study conducted at a tertiary care center in North India, aiming to enroll a minimum of 50 patients who recovered from COVID-19 pneumonia. These patients were previously hospitalized with moderate to severe disease severity as defined by the Indian Council of Medical Research (ICMR) criteria, and the assessment occurred at least three months after their discharge. Individuals who were under 18 years of age or pregnant or had any respiratory or cardiac illness in the past were excluded from the study.

Results

A total of 50 patients were included in the study for final analysis. After a three-month follow-up, 40 (80%) patients were still symptomatic. The most commonly reported symptom was exertional dyspnea in 21 (42%), dyspnea at rest in 16 (32%), and fatigue in three (6%) patients. Of the total patients, 37 (74%) covered a distance less than expected in the six-minute walk test. The mean distance covered by patients was 426.1 ± 115.01 m, in contrast to the expected mean distance of 537.22 ± 37.61 m according to standard equations for Indian males and females. A fall in oxygen saturation by more than or equal to 3% was observed in approximately 24 (48%) patients after the six-minute walk test. The mean value of fatigue and dyspnea score was 3.2 ± 1.7 (moderate score). Among patients with moderate disease during their hospital stay, a higher proportion exhibited a normal pattern on pulmonary function tests (PFT) compared to those severely affected, 23 (69.70%) versus two (11.76%), respectively.

Conclusion

The persistence of symptoms and functional limitation of activities should be anticipated in patients with COVID-19. Spirometry and 6MWT can be a valuable tool in determining the prevalence of functional limitation in recovered patients of COVID-19. It can potentially help in determining and further planning the rehabilitative measures in the management of COVID-19 survivors. It can also be concluded that it is important to have a long-term follow-up in patients with moderate to severe COVID-19.

## Introduction

Coronaviruses (CoVs) were initially considered more relevant to veterinary medicine than to human health. However, in the 21st century, their impact on human health has become evident. In 2002, severe acute respiratory syndrome (SARS) emerged, causing high mortality rates and a global health threat. A decade later, in 2012, Middle East respiratory syndrome (MERS) appeared, proving even deadlier than SARS. Toward the end of 2019, a new virus known as novel coronavirus (SARS-CoV-2) surfaced, leading to the ongoing pandemic [1].

Coronavirus disease 2019 (COVID-19) is a serious global threat, and numerous studies have been conducted to investigate the various aspects of the disease, including the host, environment, causative organism, pathophysiology, clinical features, treatment, complications, and long-term follow-up. It has a long-term impact on health, which is yet to be discovered. Studies have shown persistent residual impairment in patients. In this study, factors that are important are being studied. However, there is still much to explore about this disease.

In January 2020, a new variant of coronavirus was identified and isolated. Until July 2023, there had been a confirmed total of 767,972,961 COVID-19 cases reported worldwide, resulting in 6,950,655 deaths [2].

The primary mode of transmission for the new-variant SARS-CoV-2 is through small droplets or aerosols produced during coughing or sneezing in close contact, specifically within a distance of 2 m [3]. COVID-19 can affect any body system, but it primarily affects the respiratory system [4]. The severity of the disease is a major factor in determining the associated morbidity and mortality.

The clinical manifestations of COVID-19 infection vary widely, from asymptomatic carriage to atypical pneumonia, and can include a hyper-inflammatory phenotype or cytokine storm that leads to respiratory failure or acute respiratory distress syndrome (ARDS). The majority of ARDS patients may develop histopathological evidence of pulmonary fibrosis, and survivors may exhibit residual pulmonary fibrosis and functional impairment, indicating some degree of residual pulmonary dysfunction [5]. Pulmonary function tests (PFT), including forced expiratory volume in one second (FEV1), forced vital capacity (FVC), FEV1/FVC ratio, and a six-minute walk test (6MWT), are non-invasive and cost-effective tools for identifying any residual lung disease in post-COVID-19 patients.

Even after patients with COVID-19 test negative for the virus, a significant number of survivors continue to experience symptoms [6]. Therefore, it is crucial to understand how to manage these post-COVID-19 sequelae, which can range from mild fatigue to severe respiratory conditions requiring long-term oxygen therapy or even lung transplantation due to pulmonary fibrosis.

Spirometry involves maximal exhalation after deep inhalation, assessing lung function through indices such as FVC and FEV1. The six-minute walk test evaluates functional exercise capacity over a 30 m surface, reflecting overall exercise ability. This study aims to assess pulmonary functions and functional limitations using spirometry and a six-minute walk test (6MWT) [7].

## Materials and methods

Study design

This was an observational, prospective, and cross-sectional study performed in a tertiary care center in North India, aiming to enroll a minimum of 50 patients who recovered from COVID-19 pneumonia. These patients were previously admitted to Post Graduate Institute of Medical Sciences (PGIMS), Rohtak, with a moderate to severe disease severity as defined by the Indian Council of Medical Research (ICMR) criteria, and the assessment occurred at least three months after their discharge. The Biomedical Research Ethics Committee of Pandit Bhagwat Dayal Sharma Post Graduate Institute of Medical Sciences (PGIMS)/University of Health Sciences (UHS), Rohtak, issued approval BREC/20/TH-Gen.Medicine/15.

Patients who were reverse transcription-polymerase chain reaction (RT-PCR)-positive for COVID-19 three months back were included in the study. Patients who refused to provide consent, pregnant females, patients who are less than 18 years of age, patients who are unable to perform 6MWT, and patients with previous myocardial infarction within one month or other known cardiovascular or pulmonary disorders such as unstable angina, risk of arrhythmia, risk of cardiovascular collapse, history of pulmonary embolism, cardiomyopathy, ascending aortic aneurysm, major thoracic, and abdominal surgery or had pneumothorax, chronic bronchitis, or bronchial asthma were excluded from the study.

All the eligible patients underwent pulmonary function testing by the Morgan lung function testing machine as per the acceptability criterion of the spirometry as per the American Thoracic Society [8]. Before the procedure, the patients were advised not to smoke or vape or use a water pipe within one hour before testing, to avoid vigorous exercise within one hour before testing, and to come in loose clothing to avoid any external restrictions on lung function.

Patients were also advised not to cough during the first second of exhalation and to avoid any leak and early termination and were encouraged to do a maximal effort and not to stop before completing the full expiration. Using spirometry, FEV1, FVC, FEV1/FVC, and peak expiratory flow rate (PEFR) were recorded for all the patients after applying the acceptability criteria.

The 6MWT was conducted in the medicine ward corridor, which was 100 ft long and had a flat, straight, and hard surface. The corridor's length was marked every 3 m, and the turnaround points were indicated by cones. A starting line, marked with bright-colored tape, indicated the beginning of each 60 m lap. Patients were instructed to walk as quickly as possible on the flat, hard surface for six minutes, with permission to slow down or stop if necessary. After completing the walk, their perceived exertion, dyspnea, and fatigue were graded according to the Borg scale (Table 1).

**Table 1:** The Borg CR scale® (CR10) Scale printed with permission. The scale and full instruction can be obtained through BorgPerception AB (www.borgperception.se) ©Gunnar Borg, 1982, 1998, 2004 CR: category-ratio

Borg Scale	Perceived Exertion
0	Nothing at all
0.5	Extremely weak
1	Very weak
2	Weak
3	Moderate
5	Strong
7	Very strong
10	Extremely strong

Patients were instructed to stop if they experienced chest pain, mental confusion, lack of coordination, light-headedness, intolerable dyspnea, leg cramps or extreme leg muscle fatigue, or persistent oxygen saturation of less than 80%.

The expected distance to be covered by patients, specific for age and sex, was calculated as per reference equations for 6MWT in the healthy Indian population [9]. In Indian males, it is calculated follows: $561.022 - (2.507 \times \text{age in years}) + (1.505 \times \text{weight in kgs}) - (0.055 \times \text{height in cm}). R^2 = 0.288$. In Indian females, it is calculated follows: $30.325 - (0.809 \times \text{age in years}) - (2.074 \times \text{weight in kgs}) + (4.235 \times \text{height in cm}). R^2 = 0.272$. $R^2 = \text{coefficient of determination}$.

Statistical analysis

Data was collected by observing the distance covered, vitals before and after, and a semi-structured questionnaire self-reported by the patient. The data was entered in the Microsoft Excel (Microsoft Corp., Redmond, WA) spreadsheet, and the final analysis was done with the use of Statistical Package for Social Sciences (SPSS) version 25.0 (IBM SPSS Statistics, Armonk, NY). Descriptive statistics were performed, and the results were interpreted in frequency and percentage. Comparisons of continuous variables between groups were performed with the independent samples t-test. Comparisons between categorical variables were performed using the chi-square (χ²) test. A value of p of less than 0.05 was considered to be statistically significant.

## Results

We enrolled 50 patients out of which 34 (68%) were male and 16 (32%) were female, with a mean age of 48.2 ± 11.8 years and a mean BMI of 26.94 ± 4.2 kg/m^2^. The majority of the patients (40, 80%) continued to experience symptoms, with exertional dyspnea reported in 21 (42%), dyspnea at rest in 16 (32%), and fatigue in three (6%) patients. The baseline characteristics of the study population are detailed in Table 2.

**Table 2:** Baseline characteristics of the study population (n = 50) DM, diabetes mellitus; HTN, hypertension; COVID-19, coronavirus disease 2019

	Characteristics	n (%)
Gender	Male	34 (68%)
	Female	16 (32%)
Healthcare workers	Yes	20 (40%)
	No	30 (60%)
Occupation	Business	10 (20%)
	Service	15 (30%)
	Dependent	20 (40%)
	Others	5 (10%)
Comorbidities	None	36 (72%)
	DM	13 (26%)
	HTN	3 (6%)
Severity	Moderate	33 (66%)
	Severe	17 (34%)
COVID-19 wave	First wave	19 (38%)
	Second wave	31 (62%)
Persistent symptoms at three months	No symptoms	10 (20%)
	Dyspnea	16 (32%)
	Exertional dyspnea	21 (42%)
	Fatigue	3 (6%)

PFT revealed a normal pattern in 25 (50.00%) patients, followed by a restrictive pattern in 15 (30%) patients, a mixed pattern in six (12%) patients, and an obstructive pattern in only four (8%) patients. A total of 40 (80%) patients had a normal FEV1/FVC ratio, while 10 (20%) had a lower ratio. The mean FEV1/FVC ratio was 78.54 ± 11.18. Most patients (40, 80%) had FEV1 (percent predicted) greater than 70%, with mean values of 76.03 ± 19.59 for FEV1 (percent predicted) and 79.5 ± 19.13 for FVC (percent predicted).

Before the six-minute walk test, the mean systolic blood pressure was 118.48 ± 9.9 mmHg, and the mean diastolic blood pressure was 74.68 ± 6.67 mmHg. Following the test, there was a nonsignificant increase in mean systolic blood pressure to 126.32 ± 9.17 mmHg and mean diastolic blood pressure to 79.6 ± 6.05 mmHg. The expected mean distance for the six-minute walk test was 537.22 ± 37.61 m, but the actual mean covered distance was only 426.1 ± 115.01 m. A total of 37 (74%) patients covered a distance less than expected (p = 0.001). Oxygen saturation decreased from a mean of 97.94% ± 1.56% before the six-minute walk test to 94.28% ± 3.4% after the test. Approximately 24 (48%) patients experienced a decrease of around 3.6% in oxygen saturation.

The proportion of patients with comorbidities had a statistically significant association with severe COVID-19 during hospitalization (p = 0.004). Diabetes mellitus was the most common comorbidity seen in 13 (26%) patients, followed by hypertension in three (6.00%) patients.

**Figure 1:**
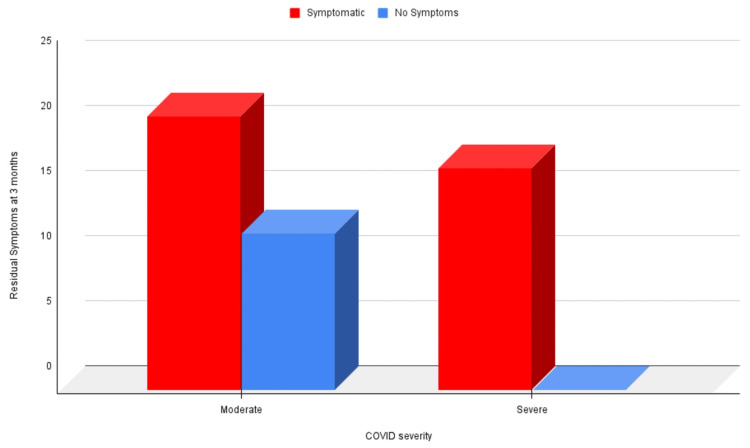
Correlation of residual symptoms at three months and COVID-19 severity at the time of hospitalization COVID-19: coronavirus disease 2019

**Figure 2:**
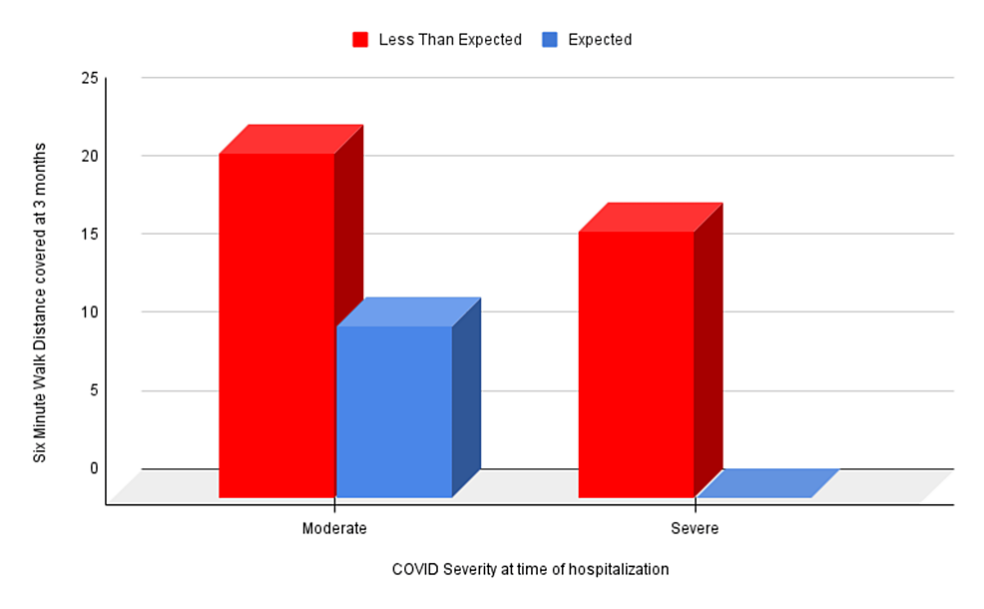
Correlation of six-minute walk distance covered at three months and COVID-19 severity at the time of hospitalization COVID-19: coronavirus disease 2019

**Figure 3:**
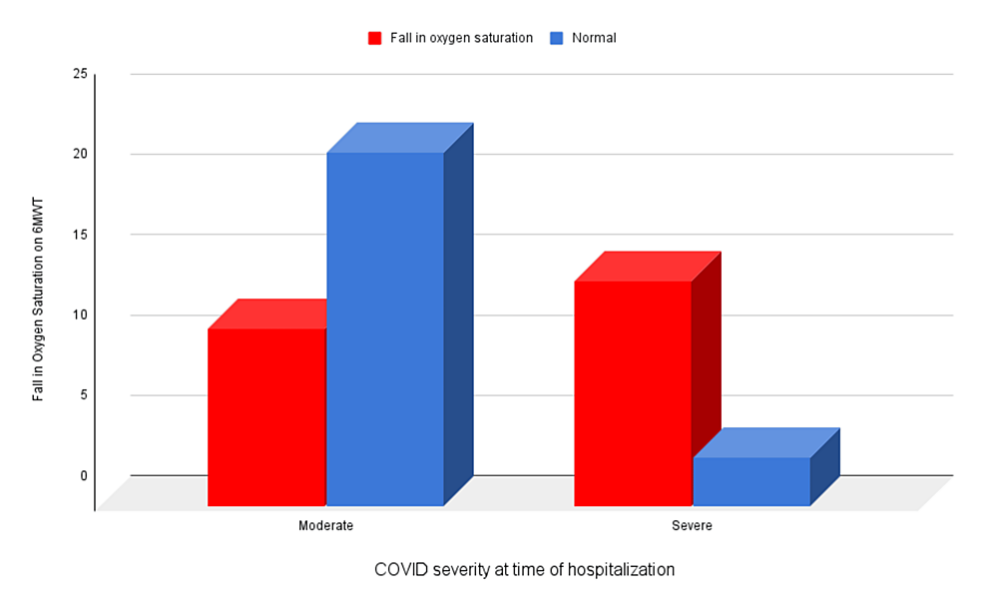
Correlation of fall in oxygen saturation on 6MWT at three months and COVID-19 severity at the time of hospitalization 6MWT, six-minute walk test; COVID-19, coronavirus disease 2019

At three months, all patients with severe COVID-19 still experienced symptoms (Figure 1) (p = 0.004), covered less distance in the six-minute walk test (Figure 2) (p = 0.009), and had a fall in oxygen saturation by 3% or more (Figure 3) (p = 0.002).

Patients with severe COVID-19 were more likely to exhibit abnormal patterns on PFT compared to those with moderate disease (p = 0.0002) (Table 3).

**Table 3:** Association of pattern on PFT at three months of follow-up with COVID-19 severity during hospitalization P-value calculated using Fisher's exact test PFT, pulmonary function test; COVID-19, coronavirus disease 2019; χ², chi-square

Type of Pattern on PFT	Moderate (n = 33)	Severe (n = 17)	Total	χ² Value	P-value
Normal pattern	23 (69.7%)	2 (11.76%)	25 (50%)	15.06	0.0002
Abnormal pattern	10 (30.30%)	15 (88.24%)	25 (50%)		
Total	33 (66%)	17 (34%)	50 (100%)		

Symptomatic patients were significantly more likely to cover a six-minute walk distance (6MWD) less than expected (p = 0.000004) and experience a decrease in oxygen saturation (p = 0.0001). Additionally, the decrease in oxygen saturation was higher in comorbid patients (p = 0.025). Furthermore, in patients with a six-minute walk distance less than expected, a decline in oxygen saturation of 3% or more was noted in 25 (64.1%) patients, which was statistically significant (p = 0.0002).

**Figure 4:**
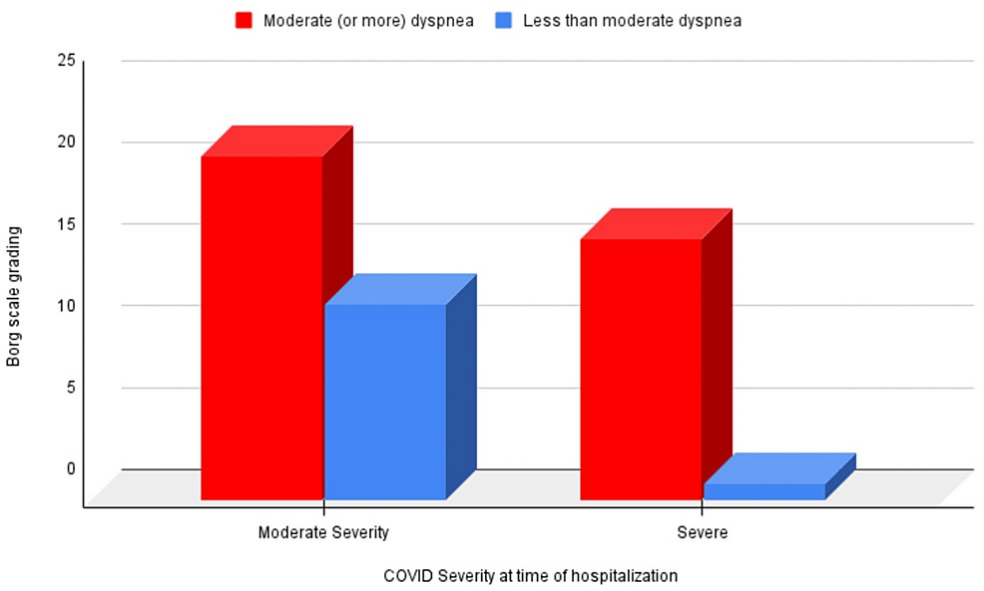
Correlation of Borg scale grading ≥3 at three months of follow-up with COVID-19 severity at the time of hospitalization COVID-19: coronavirus disease 2019

A Borg scale grading of more than or equal to 3 was common among patients with severe COVID-19 (Figure 4) (p = 0.0379). This grading was also associated with covering less than the expected distance in the six-minute walk test (p = 0.00001) and experiencing a fall in oxygen saturation by 3% or more (p = 0.00001).

The proportion of patients covering less than the expected distance in the six-minute walk test was significantly higher in individuals with abnormal PFT (Table 4) (p = 0.00003).

**Table 4:** Association of six-minute walk distance covered (meters) with abnormal PFT P-value calculated using Fisher's exact test χ², chi-square; PFT, pulmonary function test

Six-Minute Walk Distance Covered (Meters)	Normal PFT (n = 25)	Abnormal PFT (n = 25)	Total	χ² Value	P-value
Less than expected	12 (48%)	25 (100%)	37 (74%)	17.57	0.00003
Up to expected	13 (52%)	0 (0%)	13 (26%)		
Total	25 (50%)	25 (100%)	50 (100%)		

## Discussion

COVID-19 pneumonia is a viral pneumonia caused by SARS-CoV-2, first reported in in 2019. The disease has since spread globally and is a significant threat to public health. Many studies have been conducted to understand its pathophysiology, clinical features, treatment options, and long-term outcomes. However, there is still much to learn about this disease.

COVID-19 pneumonia primarily affects the respiratory system but can involve other body systems as well. The severity of the disease can vary, ranging from asymptomatic or mild cases to severe ARDS that can lead to death. Disease severity is also influenced by preexisting comorbidities, such as diabetes mellitus or chronic obstructive pulmonary disease (COPD).

Pathophysiological changes seen after COVID-19 infection include diffuse pulmonary tissue injury, cellular fiber mucous exudate, a wide spectrum of interstitial inflammatory changes, membrane thickening, pulmonary tissue damage, extracellular matrix accumulation, and interstitial fibrosis caused by lung tissue injury mediated via inflammation. Inflammatory damage to type II alveolar epithelial cells leads to a lack of surface active substance, which further leads to the collapse of small airways, decreased lung compliance, and small airway dysfunction, affecting the diffusion of respiratory gases [10].

Patients admitted to the hospital with COVID-19 pneumonia requiring oxygen support or steroid therapy often remain symptomatic even after discharge. Some people who have been infected with COVID-19 can experience long-term effects, known as long COVID-19. Long COVID-19 is broadly defined as signs, symptoms, and conditions that continue or develop after the initial COVID-19 infection. Major signs and symptoms include fatigue, dyspnea, palpitations, and insomnia. Long-term follow-up studies have shown that symptoms such as fatigue and dyspnea can persist for up to 12 weeks or longer [11].

The standard spirometry maneuver is a maximal forced exhalation (greatest effort possible) after a maximum deep inspiration (completely full lungs). Several indices can be derived from spirometry including forced vital capacity (FVC), forced expiratory volume in one second (FEV1), and FEV1/FVC. Values of FEV1 and FVC are measured in liters and are also expressed as a percentage of the predicted values for that individual. The ratio of FEV1/FVC is normally between 0.7 and 0.8 (70%-80%). Values below 0.7 are a marker of airway obstruction, except in older adults where values 0.65-0.7 may be normal as per the Global Initiative for Chronic Obstructive Lung Disease (GOLD) criteria.

The 6MWT is a simple test that requires only a 30 m or 100 ft flat, hard surface and no special equipment or training. It measures the total distance covered by the patient in six minutes, six-minute walk distance (6MWD), evaluating the integrated responses of multiple systems during exercise. The 6MWT does not provide specific information about the function of individual organs or the mechanisms of exercise limitation compared to cardiopulmonary exercise testing. Patients set their own pace and intensity during the test and may stop or rest as needed. The 6MWD may reflect functional exercise capacity for daily activities performed at sub-maximal capacity [7].

Hence, long-term follow-up is necessary to monitor patients' condition closely and take early rehabilitation measures.

In a study conducted by Chopra et al., out of the 488 patients who completed the survey for this study, 32.6% were still experiencing symptoms, with 18.9% reporting new or worsened symptoms. The most commonly reported symptom was exertional dyspnea (22.9%), followed by cough (15.4%) and persistent loss of taste and/or smell (13.1%) [12].

Similar findings were observed in a study by Carfi et al. involving 143 patients, where fatigue (53.1%), dyspnea (43.4%), joint pain (27.3%), and chest pain (21.7%) were reported as symptoms occurring 60 days after discharge. In our study, 40 (80%) of the total patients were still experiencing symptoms. The most commonly reported symptom among the patients was exertional dyspnea in 21 (42%) patients, followed by dyspnea even at rest in 16 (32%) and fatigue in three (6%) patients [2].

Previous studies have reported that diabetes increases the risk of COVID-19 severity leading to prolonged hospitalization and mortality. A study by de Almeida-Pititto et al. showed that there was a 2.3-fold increase in the risk of severity and a 2.5-fold increase in the mortality associated with COVID-19 patients with diabetes mellitus [13]. In our study, patients who had a history of diabetes mellitus had a higher proportion of severe coronavirus disease (69.23%) as compared to patients with no past history of diabetes mellitus (21.62%) (p = 0.005).

The use of non-invasive and inexpensive procedures such as PFT, which includes FEV1, FVC, FEV1/FVC ratio, and the six-minute walk test, has proven valuable in identifying residual lung disease in individuals post lung infections, including COVID-19. Several studies, including those by Truffaut et al. [14], Zhao et al. [15], and Babadi et al. [16], have demonstrated the persistent impact on lung function and other parameters in post-COVID-19 patients.

Truffaut et al. observed abnormal PFT results in 55% of patients, indicating a restrictive pattern and altered diffusing capacity of the lungs for carbon monoxide (DLCO). Patients continued to experience disability in lung function, reduced exercise capacity, reduced quality of life, and exertional dyspnea even three months after discharge [14]. Similarly, Zhao et al.'s study on non-critically ill patients in China revealed lung function anomalies in 25.54% of patients three months after discharge, with abnormalities in total lung capacity (TLC), FEV1, FVC, DLCO, and small airway dysfunction. This study highlighted significant abnormalities persisting beyond the resolution of respiratory symptoms [15]. Babadi et al.'s study on severe COVID-19 pneumonia patients found 3.1% with an obstructive pattern, 40.63% with a restrictive pattern, and 6.25% with a mixed pattern. These findings underscore the diverse pulmonary function outcomes in individuals recovering from severe COVID-19 [16].

Our study aligns with these findings, indicating a mean FEV1/FVC of 78.54 ± 11.18. The mean values of FEV1 (percent predicted) and FVC (percent predicted) were 76.03 ± 19.59 and 79.5 ± 19.13, respectively. In our study, the majority of patients had a normal or higher FEV1/FVC (80%), while 20% exhibited FEV1 of less than 70%. Notably, 50% of our patients showed residual disease on PFT, of which 30% exhibited a restrictive pattern, 8% had an obstructive pattern, and 12% had a mixed pattern on PFT.

In a study conducted by Huang et al., the mean distance covered during the 6MWT for all patients was found to be 561.97 m (±45.29 m). Patients classified as severe cases demonstrated a shorter six-minute walking distance (517.43 m) compared to non-severe cases. Moreover, the 6MWD in severe cases reached only 88.4% of the predicted values, significantly lower than in non-severe cases. These findings suggest a reduced exercise capacity following recovery from COVID-19 [17].

Fernández-de-Las-Peñas et al. conducted a study in Spain involving 1,142 post-COVID-19 patients who were evaluated seven months after hospitalization. The study results revealed that 45% of the patients reported functional limitations in their daily living activities, as assessed by the Functional Impairment Checklist [18]. In our study, we assessed fatigue and dyspnea using the Borg scale. It is commonly used to measure perceived exertion or dyspnea (shortness of breath) on a scale from 0 to 10. The mean score for fatigue and dyspnea was 3.2 ± 1.7. Moreover, a Borg scale grading of more than or equal to 3, which indicated significant discomfort or difficulty breathing, was seen in the majority of patients with severe COVID-19 (94.1%) and symptomatic patients (89.74%). These findings highlight the association between decreased exercise capacity and the perception of increased dyspnea as measured by the Borg scale grading.

A correlation between COVID-19 severity and functional limitation was also seen. One hundred percent of patients who had severe COVID-19 were still having symptoms at three months and covered a six-minute walk distance less than expected. A similar association was seen in a study by Pant et al. [19].

The present study evaluated patients' condition three months after discharge using the six-minute walk test and Borg scale. It evaluated the impact of associated comorbidities and COVID-19 severity at the time of hospitalization on clinical outcomes at three months of follow-up. The study limitations include a smaller sample size and a shorter duration of follow-up. To reinforce the study's findings, larger sample sizes and diverse populations need to be studied for longer periods, such as six months or a year.

## Conclusions

COVID-19 is a serious global threat that can affect any body system, but it primarily affects the respiratory system. Disease severity is a significant predictor of associated morbidity and mortality. The greater severity of COVID-19 during hospitalization was associated with residual pulmonary deficits at three months, as evidenced by reduced exercise capacity during the six-minute walk test (6MWT) and Borg scale. Moreover, the findings of this study indicate that patients with comorbidities experienced a more severe form of COVID-19. Among comorbid patients, COVID-19 was particularly severe in those with diabetes mellitus.

In light of these study findings, it can be concluded that patients in the moderate to severe category exhibit residual lung dysfunction, particularly in terms of reduced exercise capacity at three months of follow-up. Therefore, long-term follow-up is crucial even after discharge from the hospital. Also, PFT, 6MWT, and the Borg scale are good and sensitive tests to assess the functional status of post-COVID-19 patients.
